# The Uptake of and Access to Expanded Program for Immunization Services Among Children Aged 0 to 12 Months During the COVID‐19 Pandemic in Free State Province, South Africa: A Mixed Study

**DOI:** 10.1002/hsr2.73470

**Published:** 2026-11-10

**Authors:** Abebe Sorsa Badacho, Nomvuyiseko Maduna, Juliet Nyasulu

**Affiliations:** ^1^ Division of Health Systems and Public Health, Department of Global Health, Faculty of Health Science and Medicine Stellenbosch University Cape Town South Africa; ^2^ School of Public Health Wolaita Sodo University Wolaita Sodo Ethiopia; ^3^ School of Public Health, Faculty of Health Sciences University of the Witwatersrand Johannesburg South Africa; ^4^ Health Systems Strengthening, AFRIQUIP Johannesburg South Africa

**Keywords:** child immunization, Expanded Programme on Immunization, health service access, health system resilience, mixed‐methods study

## Abstract

**Background:**

The COVID‐19 pandemic disrupted essential health services, including routine childhood immunization. This study assessed immunization uptake and access among children aged 0–12 months and explored the effects of COVID‐19 on Expanded Programme on Immunization (EPI) service delivery in the Free State Province, South Africa.

**Methods:**

A mixed‐methods exploratory study was conducted. Quantitative data were obtained from the District Health Information System (DHIS) and used to compare EPI uptake during the 12 months before and 12 months after the onset of COVID‐19. Data were analyzed using Wilcoxon rank‐sum and Kruskal–Wallis tests. Qualitative data were collected through unstructured interviews with provincial and district EPI and Maternal and Child Health managers, and were analyzed thematically using the World Health Organization health systems building blocks framework.

**Results:**

Although immunization uptake among children under 1 year declined following the onset of COVID‐19, the reductions were not statistically significant (*p* > 0.05). Qualitative findings indicated that the pandemic increased pressure on the health system, adversely affecting access to and delivery of primary healthcare services, including immunization. Key challenges included staff shortages, inadequate funding, weak emergency preparedness, limitations in health information systems, and shortcomings in leadership and governance. Participants reported that these health system weaknesses existed before the pandemic but were intensified during the COVID‐19 period.

**Conclusion:**

Routine childhood immunization services in the Free State Province were largely maintained during the COVID‐19 pandemic despite increased pressure on the health system. While the pandemic exposed and exacerbated existing health system weaknesses, it also demonstrated the resilience of immunization services. Strengthening health system preparedness, workforce capacity, information systems, and governance will be essential for sustaining immunization services during future emergencies.

## 1 Background

The COVID‐19 pandemic disrupted essential health services worldwide, including routine childhood immunization. Following the emergence of SARS‐CoV‐2, the World Health Organization (WHO) declared COVID‐19 a Public Health Emergency of International Concern and subsequently a pandemic [1, 2]. In South Africa, the first confirmed case was reported on March 5, 2020, followed by public health measures including a national lockdown and a risk‐adjusted alert system to limit transmission [3].

Routine childhood immunization remains one of the most cost‐effective public health interventions for preventing morbidity and mortality from vaccine‐preventable diseases [4, 5]. Childhood immunization contributes significantly to universal health coverage (UHC), the sustainable development goals (SDGs), and economic productivity [6, 7]. Despite progress in vaccination coverage globally, persistent inequalities and health system challenges continue to affect immunization uptake in many low‐ and middle‐income countries (LMICs) [8, 9, 10].

Prior to the pandemic, barriers to immunization included shortages of healthcare workers, vaccine stock‐outs, inadequate infrastructure, weak health information systems, and socioeconomic inequalities [8, 9, 10, 11]. These existing vulnerabilities raised concerns regarding the ability of health systems to sustain routine immunization services during a major public health emergency.

International evidence indicates that COVID‐19 substantially disrupted routine childhood immunization services. WHO and UNICEF reported the largest sustained decline in childhood vaccination coverage in approximately three decades [12, 13]. Global modeling studies estimated significant reductions in routine vaccine coverage during 2020, increasing the risk of outbreaks of vaccine‐preventable diseases [14]. Shet et al. further documented widespread disruptions to immunization services across 170 countries and territories [15].

Studies from LMICs have demonstrated considerable declines in immunization service utilization during the pandemic. Significant disruptions were reported in Pakistan, Ethiopia, Kenya, Rwanda, and several other African countries due to lockdowns, mobility restrictions, fear of infection, staff redeployment, and interruptions to outreach services [16, 17, 18, 19, 20]. Evidence from high‐income settings also suggests that the impact of the COVID‐19 pandemic on childhood immunization uptake was not uniform, with declines reported in England but relatively stable uptake observed in Scotland during the pandemic [21].

Understanding the impact of COVID‐19 on immunization services in the Free State Province is important for strengthening health‐system resilience and preparedness for future public health emergencies. A mixed‐methods approach was therefore employed to provide a comprehensive understanding of both the magnitude of changes in childhood immunization uptake and the health‐system factors influencing service delivery during the pandemic. While quantitative DHIS data enabled assessment of trends in immunization uptake before and during COVID‐19, qualitative interviews with program managers provided insights into the contextual, operational, and health‐system challenges underlying these trends. Guided by the WHO Health Systems Building Blocks Framework, the integration of quantitative and qualitative findings allowed for a more comprehensive assessment of the pandemic's effects on EPI service delivery and helped identify strategies to sustain immunization services during future emergencies [22].

## 2 Methods

### 2.1 Study Design

An explanatory mixed‐methods study was conducted using a descriptive exploratory design. The quantitative component involved secondary analysis of routine immunization data to assess changes in Expanded Programme on Immunization (EPI) uptake before and during the COVID‐19 pandemic. The qualitative component subsequently explored health managers' experiences and perceptions to explain quantitative findings and identify health‐system factors influencing EPI service delivery. Quantitative and qualitative data were analyzed independently and integrated during interpretation to provide a comprehensive understanding of the effects of the COVID‐19 pandemic on immunization services.

### 2.2 Study Setting and Population

The study was conducted in the Free State Province, South Africa, comprising five health districts. The quantitative component used routine immunization data for children aged 0–12 months who received immunization services at public health facilities and whose vaccinations were reported through the District Health Information System (DHIS). Data from the 12 months preceding the pandemic (March 2019–February 2020) were compared with data from the 12 months following the emergence of COVID‐19 (March 2020–February 2021).

For the qualitative component, key informants included provincial and district managers responsible for the EPI and Maternal, Child and Women's Health (MCWH) programs. Participants were purposively selected based on their involvement in EPI program planning, implementation, and monitoring during the pandemic.

### 2.3 Sampling and Recruitment

For the quantitative component, all available routine immunization records reported through the DHIS for children aged 0–12 months who received immunization services at public health facilities in the five districts during the study period were included in the analysis. The DHIS contains aggregate immunization service data and does not provide records for all children residing in the districts; therefore, children who did not attend health facilities for immunization could not be identified. No sampling was undertaken, as all available records meeting the inclusion criteria were analyzed.

For the qualitative component, purposive sampling was used to recruit one provincial program manager and five district program managers. Managers directly involved in EPI or MCWH program implementation during the study period were eligible to participate.

### 2.4 Data Collection

#### 2.4.1 Quantitative Data

Secondary aggregate immunization data were obtained from the National Department of Health's District Health Information System (DHIS). The dataset comprised routinely reported immunization service indicators from public health facilities across the five districts. All available records for children under 1 year of age who received immunization services during the study period were included in the analysis. Immunization indicators for children under 1 year of age were extracted for the two study periods. Indicators included BCG coverage, DTaP‐IPV/Hib coverage, Hepatitis B coverage, measles vaccination coverage, oral polio vaccine coverage, rotavirus vaccination coverage, immunization coverage under 1 year, and immunization drop‐out rates.

#### 2.4.2 Qualitative Data

Semi‐structured key informant interviews were conducted virtually using Microsoft Teams. An interview guide informed by the WHO Health Systems Building Blocks Framework was used to explore participants' experiences of EPI service delivery during the COVID‐19 pandemic, perceived barriers to immunization access, health‐system challenges, and strategies employed to maintain services. Interviews were audio‐recorded, transcribed verbatim, and anonymized prior to analysis. The interview guide was piloted with program managers from outside the study province to improve clarity and consistency.

### 2.5 Data Analysis

#### 2.5.1 Quantitative Analysis

Descriptive statistics were used to summarize immunization uptake across the study periods. Immunization trends before and during the COVID‐19 pandemic were compared using non‐parametric statistical tests because data were not normally distributed. The Wilcoxon rank‐sum test was used to compare immunization uptake between the two periods, while the Kruskal–Wallis test assessed differences across districts. Statistical analyses were conducted at a significance level of 5%.

#### 2.5.2 Qualitative Analysis

Qualitative data were analyzed using thematic analysis. Interview transcripts were read repeatedly to facilitate familiarization with the data. Coding was conducted using both deductive and inductive approaches. Initial coding was guided by the WHO Health Systems Building Blocks Framework, covering service delivery, health workforce, health information systems, medical products and technologies, health financing, and leadership and governance. Additional themes emerging from the data were incorporated inductively. Codes were grouped into categories and themes, which were reviewed, refined, and interpreted to explain the quantitative findings.

#### 2.5.3 Integration of Quantitative and Qualitative Findings

Findings from both components were integrated during interpretation using triangulation. Quantitative results describing changes in immunization uptake were compared with qualitative findings on health‐system experiences and challenges to provide a comprehensive understanding of the impact of COVID‐19 on EPI service delivery and access in the Free State Province.

## 3 Results

### 3.1 Quantitative Findings

#### 3.1.1 Changes in Immunization Uptake Before and During the COVID‐19 Pandemic

A comparison of routine immunization data for children aged 0–12 months during the 12 months before (March 2019–February 2020) and 12 months after (March 2020–February 2021) the emergence of COVID‐19 showed a general decline in immunization uptake across most indicators (Table 2). However, none of the observed changes were statistically significant (all *p* > 0.05).

The largest declines were observed for the third dose of DTaP‐IPV‐Hib‐HBV‐Hexavalent vaccine (difference = −2029), measles second dose (difference = −1730), immunization coverage among children fully immunized under 1 year (difference = −1075), and PCV coverage (difference = −1237). Smaller declines were observed for BCG vaccination (difference = −411). In contrast, increases were observed for the first dose of DTaP‐IPV‐Hib‐HBV‐Hexavalent vaccine (difference = 1616), measles first dose (difference = 1086), and rotavirus second dose (difference = 261).

Overall, although immunization uptake decreased for most indicators following the emergence of COVID‐19, the reductions were modest and did not reach statistical significance, suggesting that routine immunization services were largely maintained during the study period. Immunization access of children aged 0–12 months, 12 months before and 12 months after the emergence of the COVID‐19 pandemic in Free State, South Africa (Table 1).

**Table 1:** Rank sum test of 0–12 months old children immunized before and after the COVID‐19 first case in Free State Province in South Africa.

Indicator	Rank sum test of children immunized (before)	Rank sum test children immunized (after)	Difference in rank sum of children immunized (after–before)	*p* value
BCG dose	66,126	65,715	−411	0.2636
DTaP‐IPV‐Hib‐HBV‐Hexavalent 1st	69,020	70,636	1616	0.6448
DTaP‐IPV‐Hib‐HBV‐Hexavalent 3rd	708,425	68,813.5	−2029	0.5627
Immunized fully under 1 year new	70,365.5	69,290.5	−1075	0.7591
Measles 1st dose	69,285	70,371	1086	0.7567
Measles 2nd dose	70,693	68,963	−1730	0.6217
PCV	70,446.5	69,209.5	−1237	0.7242
RV 2nd dose under 1 year	69,697.5	69,958.5	261	0.9406

#### 3.1.2 District‐Level Differences in Immunization Uptake

District‐level analysis revealed heterogeneous patterns of immunization uptake across the five districts of the Free State Province (Table 2). Most districts experienced fluctuations in immunization coverage across different vaccine indicators; however, few statistically significant changes were observed.

**Table 2:** Rank sum test of 0–12 months old children immunized before and after the COVID‐19 first case in the five districts of Free State Province in South Africa.

District	Analysis	Indicator							
		BCG dose	DTaP‐IPV‐ Hib‐HBV (Hexavalent) _1st_	DTaP‐IPV‐ Hib‐HBV (Hexavalent) 3rd	Immunized fully under 1 year new	Measles 1st dose	Measles 2nd dose	PCV	RV 2nd dose under 1 year
Fezile Dabi District	Number immunized (before)	2190	2362	2478	2547	2421	2672	2530	2465
	Number immunized (after)	2466	2295	2178	2109	2236	1985	2127	2191
	Difference (after–before)	−276	−67	−300	−440	−185	−687	−403	274
	*P*‐value	0.3119	0.8061	0.2716	0.1085	0.4978	0.0118	0.1398	0.3154
Lejweleputswa District	Number immunized (before)	3144	3472	3665	3673	3600	3651	3705	3686
	Number immunized (after)	3072	3788	3595	3588	3660	3609	3556	3575
	Difference (after–before)	−72	316	−70	−85	60	−42	−149	−111
	*P*‐value	0.1702	0.4069	0.8542	0.8235	0.8749	0.9122	0.6958	0.7708
Mangaung Metro	Number immunized (before)	2032	2285	2368	2350	2306	2353	2338	2328
	Number immunized (after)	2063	2372	2288	2306	2351	2304	2318	2329
	Difference (after–before)	31	87	−80	−44	45	−49	−20	1
	*P*‐value	0.3273	0.7499	0.7694	0.8719	0.8690	0.8575	0.9416	0.9971
Thabo Mofutsanyane District	Number immunized (before)	5434	5256	5433	5228	5075	5154	5193	5152
	Number immunized (after)	5007	5185	5008	5213	5365	5287	5247	5288
	Difference (after–before)	−427	−71	−425	−15	290	133	54	136
	*P*‐value	0.3936	0.8872	0.3958	0.9761	0.5623	0.7905	0.9141	0.7858
Xhariep District	Number immunized (before)	1314	1212	1229	1224	1229	1350	1235	1177
	Number immunized (after)	1314	1416	1399	1404	1400	1279	1394	1451
	Difference (after–before)	0	204	170	180	171	−71	159	274
	*P*‐value	1.000	0.2503	0.3381	0.3104	0.3353	0.6890	0.3702	0.1226

Fezile Dabi District recorded the most pronounced reductions across several indicators following the emergence of COVID‐19, including declines in BCG, DTaP‐IPV‐Hib‐HBV‐Hexavalent third dose, fully immunized children under 1 year, measles first dose, measles second dose, and PCV coverage. Among all comparisons conducted, only the decline in measles second‐dose coverage was statistically significant (*p* < 0.05).

Lejweleputswa District demonstrated relatively stable immunization performance, with small increases in DTaP‐IPV‐Hib‐HBV‐Hexavalent first‐dose coverage and measles first‐dose coverage, while slight declines were observed across the remaining indicators. None of these changes was statistically significant.

Similarly, Mangaung Metropolitan Municipality showed only minor increases and decreases across immunization indicators, with no statistically significant differences between the pre‐ and post‐COVID‐19 periods.

In Thabo Mofutsanyana District, reductions were observed in BCG, DTaP‐IPV‐Hib‐HBV‐Hexavalent first‐dose coverage and third‐dose coverage, while modest increases were noted for measles first dose, measles second dose, PCV, and rotavirus second dose. None of these changes reached statistical significance.

Xhariep District demonstrated the most favorable pattern, with increases observed in nearly all immunization indicators following the emergence of COVID‐19. Measles second‐dose coverage was the only indicator that declined slightly. However, none of the observed changes were statistically significant.

Overall, the district‐level findings indicate that the impact of COVID‐19 on routine immunization uptake varied across districts. Despite some declines in vaccine uptake, particularly in Fezile Dabi District, routine immunization services remained relatively resilient across the province, with only one statistically significant reduction identified. Immunization access of children aged 0–12 months, 12 months before and 12 months after the emergence of the COVID‐19 pandemic by district in Free State, South Africa.

### 3.2 Qualitative Findings

To complement the quantitative findings and provide a deeper understanding of the factors influencing immunization service delivery during the COVID‐19 pandemic, six key informant interviews were conducted with provincial and district managers responsible for the Expanded Programme on Immunization (EPI) and Maternal, Child and Women's Health (MCWH) programs. Participants had more than 10 years of public health experience and had served in their current management positions for at least 5 years.

Thematic analysis identified six interrelated themes aligned with the WHO Health Systems Building Blocks framework, together with an additional cross‐cutting theme relating to Risk Communication and Community Engagement. Collectively, the findings suggest that COVID‐19 did not create entirely new challenges for EPI service delivery; rather, it exposed and amplified pre‐existing health system weaknesses while prompting adaptive responses that helped sustain immunization services.

#### 3.2.1 Theme 1: Disruptions to Service Delivery Reduced Access to Immunization Services

Service delivery disruptions emerged as one of the most prominent consequences of the COVID‐19 pandemic. Participants described how movement restrictions, fear of infection, suspension of outreach activities, school closures, and temporary facility closures collectively reduced caregivers' access to immunization services. These disruptions were particularly evident during the strict lockdown periods when uncertainty surrounding COVID‐19 and public health restrictions discouraged healthcare utilization.

One district manager described how lockdown restrictions and fear of infection interacted to reduce attendance at health facilities:

Remember that the police were all over, and the army as well. Parents and caregivers were trying to comply with lockdown regulations and many were afraid of being stopped or questioned when travelling. More than that, people were scared of catching COVID‐19 if they visited health facilities. During the first two quarters, around April to September, our immunization coverage really went down. Mothers simply did not come to the facilities because they were staying indoors and avoiding places where they thought they could become infected.

(Manager 1)

This finding suggests that reductions in immunization uptake were driven not only by formal restrictions on movement but also by perceptions of risk and fear associated with healthcare facilities during the pandemic.

Participants further explained that interruptions to community outreach services weakened the health system's ability to reach children who depended on mobile services and routine community tracing mechanisms.

The community health workers who usually identify children who have defaulted on immunization were shifted to larger COVID‐19 sites and vaccination centres. Schools and crèches were closed, making it difficult to find children under one roof. Normally, it is easier when children are gathered in schools or early childhood centres because defaulters can be identified quickly. However, during COVID‐19, children were scattered throughout communities and parents were reluctant to allow health workers into their homes. This made tracing and follow‐up much more difficult.

(Manager 2)

The suspension of outreach activities therefore represented a critical loss of an important service‐delivery platform, particularly for vulnerable and hard‐to‐reach populations.

Another service delivery challenge identified by participants was the diversion of attention and resources toward the COVID‐19 response.

At first, many nurses were removed from facilities to support COVID‐19 activities. Staff were involved in screening, testing, contact tracing, investigations and later vaccination campaigns. When schools reopened, the school health teams and mobile teams were also redirected to COVID‐19 work. As a result, routine services, including immunization, had fewer staff available and service delivery became more difficult.

(Manager 3)

Participants emphasized that while COVID‐19 created new demands, it also exposed weaknesses in routine service organization that already existed before the pandemic. For example, some facilities continued to provide immunizations on selected days despite national policy advocating that every day should be an immunization day. Managers felt that these practices amplified missed opportunities for vaccination during periods of restricted access.

Despite these challenges, participants described several adaptive strategies that helped restore service utilization once restrictions eased. Catch‐up campaigns, door‐to‐door tracing, and targeted community outreach were widely viewed as effective interventions.

We developed a catch‐up plan because we knew many children had missed their vaccines during lockdown. It eventually became part of our annual planning. We started going door‐to‐door and engaging directly with communities to identify children who had not attended the clinic. It became clear that many parents were afraid of coming to facilities rather than unwilling to immunize their children. Once we actively traced them, immunization uptake started improving again.

(Manager 6)

Overall, the data indicate that service delivery disruptions during COVID‐19 were multifactorial, arising from mobility restrictions, public fear, suspension of outreach services, and diversion of healthcare resources. However, the implementation of catch‐up campaigns and community‐based tracing strategies demonstrated the health system's capacity to adapt and recover from service interruptions.

#### 3.2.2 Theme 2: Health Workforce Constraints Reduced the Capacity to Deliver Routine Immunization Services

Health workforce challenges emerged as one of the most significant mechanisms through which COVID‐19 affected EPI service delivery. Participants described how pre‐existing staff shortages were exacerbated by illness, deaths among healthcare workers, quarantine requirements, and redeployment to COVID‐19 activities. As a result, routine child health services often had to compete with pandemic response activities for the same limited workforce.

One manager reflected on the cumulative impact of workforce attrition and increased workload:

With staff getting sick, staff getting infected, staff that passed on, it really affected our primary health care services in a great way. Staff were not replaced when they left or passed away, and those who remained had to absorb the workload. You would go to a facility and find only two professional nurses on duty. One nurse would be responsible for COVID testing, putting on and taking off protective equipment, while at the same time attending to patients with acute conditions, chronic care, maternal health, and child health services. COVID not only took our staff away physically, but it also affected staff morale because people were exhausted and overwhelmed.

(Manager 2)

Participants consistently emphasized that workforce shortages existed prior to COVID‐19 but became considerably more visible during the pandemic. Staff redeployment to testing, contact tracing, case investigations, and vaccination campaigns further weakened the ability of facilities to maintain routine immunization services.

Our mobile services were really badly affected because most of the nurses assigned to mobile clinics were moved to COVID‐related work. They were testing, swabbing, conducting case investigations, and following up contacts for extended periods. The pandemic exposed what was already a challenge before COVID, which was that there were simply not enough health workers to provide all the services needed.

(Manager 4)

These findings suggest that workforce resilience was a critical determinant of immunization program performance during the pandemic.

#### 3.2.3 Theme 3: Health Information System Weaknesses Limited Effective Monitoring of Immunization Services

Participants described how weaknesses in health information systems affected the ability to monitor program performance during the pandemic. Reduced availability of data capturers, staff rotation arrangements, increased workload, and inconsistent recording practices created delays and gaps in reporting.

Importantly, managers argued that some reported declines in immunization coverage reflected deficiencies in recording and reporting rather than actual interruptions in vaccination service delivery.

The challenge was not always that children were not vaccinated. In many instances, the problem was that data were not captured correctly or on time. Data capturers were working on alternate days because of COVID restrictions, so backlogs developed. In some facilities, nurses continued providing vaccinations and recording them in clinical records, but the information was not transferred into the reporting system promptly. When we later conducted facility visits, we found evidence that vaccines had been administered but had not been entered into the system. In some cases, staff did not know that BCG and OPV given at birth also had to be reported, resulting in what looked like declines in performance.

(Manager 6)

Participants also noted that the pandemic generated important learning opportunities around data quality and information use.

The pandemic taught us the importance of monitoring and supervision. We realized that problems were often being detected by district teams instead of the facilities themselves. It highlighted the need for stronger routine monitoring, more frequent review of information, and better accountability around reporting systems.

(Manager 6)

Overall, respondents viewed robust health information systems as essential for maintaining continuity of immunization services during emergencies.

#### 3.2.4 Theme 4: Vaccine Availability Was Maintained Despite Local Supply Chain Challenges

Unlike workforce and service delivery challenges, participants generally reported that vaccine availability remained stable throughout the pandemic. Managers attributed this to strong provincial logistics systems, central distribution mechanisms, and active stock monitoring processes.

Our vaccines at the medical depot were always available. We never really experienced major shortages at provincial level. The challenge was more at facility level, where stock management and reporting were not always optimal. Sometimes facilities reported stock too late, sometimes deliveries were delayed because of movement restrictions, and occasionally short‐dated stock was received. But overall, vaccine availability itself was not the major problem.

(Manager 1)

Managers further explained how local shortages were mitigated through collaboration between facilities.

Whenever a facility reported low stock, the system allowed us to quickly identify where vaccines were available elsewhere. Stock could then be moved between facilities. Although there were isolated incidents of stock shortages, these were usually resolved quickly and did not become prolonged disruptions to service delivery.

(Manager 4)

These findings suggest that supply chain resilience played an important role in protecting immunization services during the pandemic.

#### 3.2.5 Theme 5: Leadership and Governance Influenced Health System Resilience

Leadership and governance were identified as both strengths and weaknesses within the pandemic response. Participants praised existing partnerships with private healthcare providers and development partners but highlighted shortcomings in communication, coordination, and emergency preparedness.

Managers described how uncertainty and delayed communication during the early stages of the pandemic affected service delivery.

One of the biggest challenges was communication. In the beginning, it felt like only people at the top levels knew what was happening. Information did not always reach district and facility staff quickly enough, and there was uncertainty about what was expected. During an emergency, communication must reach every level of the system, including the lowest levels, because those are the people delivering services directly to communities.

(Manager 3)

Participants repeatedly reflected on the absence of clear emergency preparedness plans.

COVID taught us that we were not prepared for a public health emergency of this scale. Many decisions had to be made as the situation unfolded rather than following established contingency plans. Looking ahead, health systems need emergency preparedness strategies that can be activated immediately, whether the crisis is a pandemic, flood, outbreak, or another disaster.

(Manager 3)

The findings indicate that governance capacity, preparedness planning, and communication systems are critical components of health‐system resilience.

#### 3.2.6 Theme 6: Inadequate Infrastructure and Digital Resources Constrained Program Performance

Although financial constraints were not widely discussed, managers consistently highlighted shortages of information technology infrastructure required for routine data management.

The challenge was often not the people themselves but the tools available to them. When there was no data, a modem was not working, a computer failed, or internet connectivity was poor, data capturing became delayed. These issues existed before COVID, but they became more noticeable when the system increasingly relied on digital reporting and virtual communication.

(Manager 3)

Participants argued that investment in digital infrastructure would strengthen program monitoring and support continuity during future emergencies.

### 3.3 Cross‐Cutting Theme: Effective Risk Communication and Community Engagement Can Sustain Immunization Uptake During Emergencies

Risk communication and community engagement emerged as a cross‐cutting theme that influenced several other building blocks. Participants reported that confusion, misinformation, and poorly coordinated messaging contributed to fear among caregivers and reduced healthcare utilization.

One manager reflected on how public communication regarding COVID‐19 vaccination unintentionally affected childhood immunization services:

Parents often heard messages about vaccination and immediately associated them with COVID vaccines. They worried that when they brought their children to the clinic, the child would receive a COVID vaccine instead of routine childhood immunization. We learned that language matters. The difference between saying ‘vaccination’ and ‘childhood immunization’ seems small, but it created confusion. During future emergencies, communication must be carefully designed and harmonized so that communities understand exactly what services are available and why they remain important.

(Manager 3)

Participants also emphasized that stronger community engagement, health education, and visibility of EPI services could help maintain service utilization during future outbreaks.

If communities are informed, they will continue to use services even during an emergency. Immunization messages should begin during antenatal care, continue in schools, community centres, and social support points. We need continuous promotion of immunization, not only when there is a crisis. COVID showed us that communication and community engagement are not optional; they are essential parts of maintaining health services.

(Manager 6)

## 4 Discussion

This study assessed the effect of the COVID‐19 pandemic on the uptake and access to Expanded Programme on Immunization (EPI) services among children aged 0–12 months in the Free State Province, South Africa. The quantitative findings showed a decrease in immunization uptake from the 12 months preceding the pandemic to the 12 months following its emergence, with no statistically significant difference. However, variations in immunization performance were observed across districts, with a significant decline in measles second‐dose uptake identified only in Fezile Dabi District.

The qualitative findings provided important contextual explanations for these results. Although immunization uptake remained relatively stable, program managers reported substantial disruptions to service delivery, workforce availability, information systems, communication processes, and emergency preparedness. Overall, the findings suggest that COVID‐19 did not create entirely new challenges for EPI service delivery but rather exposed and intensified pre‐existing weaknesses within the health system. Similar observations have been reported in South Africa and other low‐ and middle‐income countries (LMICs), where the pandemic amplified existing vulnerabilities rather than creating entirely new ones [21, 22].

### 4.1 Immunization Uptake Before and During the COVID‐19 Pandemic

The quantitative analysis showed no significant differences in overall childhood immunization uptake between the pre‐pandemic and pandemic periods. This finding contrasts with global evidence reporting significant declines in routine immunization coverage during COVID‐19. Global analyses estimated substantial disruptions in childhood vaccination services across multiple countries, largely due to movement restrictions, service interruptions, and caregiver fears of acquiring infection [14, 15, 16, 17, 18, 19, 20].

The relatively stable immunization performance observed in the Free State Province may be partly explained by the province's immunization context prior to COVID‐19. The 2019 EPI Coverage Survey reported that only 76.7% of children were fully immunized and 80.7% had received vaccines scheduled up to 12 months, indicating that immunization coverage was already below the national target before the pandemic [23]. Similar findings were reported by the MAL‐ED study, which identified persistent challenges in achieving optimal immunization coverage in LMIC settings [24].

Program managers further reported that implementation of national and provincial catch‐up immunization campaigns coincided with the pandemic period. These campaigns may have mitigated potential declines in vaccination coverage and contributed to the maintenance of routine services [25]. Consistent with this finding, WHO guidance emphasized catch‐up vaccination activities as a critical strategy for recovering immunization losses associated with COVID‐19 disruptions [15, 26].

Although provincial‐level uptake remained largely stable, the significant decline in measles second‐dose coverage observed in Fezile Dabi District deserves attention. Measles vaccination coverage was already below the recommended target before the emergence of COVID‐19, suggesting that the pandemic exacerbated existing program weaknesses rather than independently causing service deterioration [23]. Similar concerns about missed opportunities for vaccination and persistently suboptimal coverage have been reported elsewhere in South Africa [27, 28].

### 4.2 Effects of COVID‐19 on EPI Services

#### 4.2.1 Service Delivery

Service delivery emerged as the health‐system building block most visibly affected by the pandemic. Program managers described reduced healthcare utilization resulting from lockdown restrictions, fear of COVID‐19 infection, temporary facility closures, suspension of community outreach activities, and school closures.

These findings are consistent with international evidence demonstrating that mobility restrictions and fear of infection contributed to declines in healthcare utilization during the pandemic [15, 16, 17, 18, 19, 20, 21]. In South Africa, reduced attendance at primary healthcare facilities and interruptions in maternal and child health services were similarly reported during lockdown periods [29, 30, 31].

A particularly important finding was the disruption of community‐based activities. Community health workers (CHWs) were unable to conduct outreach visits, school health activities, or defaulter tracing to the same extent as before the pandemic. Similar findings have been reported in other South African settings, where interruptions in outreach activities reduced opportunities to identify children who had missed scheduled vaccinations [32, 33].

Despite these disruptions, participants highlighted the successful implementation of catch‐up campaigns and community tracing initiatives following the relaxation of restrictions. These recovery strategies likely contributed to the limited impact observed in immunization uptake.

#### 4.2.2 Health Workforce

Health workforce shortages represented one of the most important mechanisms through which COVID‐19 affected EPI service delivery. Respondents reported staff illness, quarantine requirements, deaths among healthcare workers, and redeployment of personnel to COVID‐19‐related activities, including screening, testing, contact tracing, and vaccination.

These findings are consistent with reports from Africa and other LMICs, where workforce disruptions significantly affected essential health‐service delivery [11, 20]. Participants emphasized that staffing shortages existed before the pandemic but became more pronounced during COVID‐19. Similar workforce‐related challenges have been documented in South Africa, particularly in resource‐constrained settings, where shortages contribute to missed opportunities for vaccination and reduced service quality [22, 27, 28, 29].

The findings underscore the interdependence of health‐system building blocks, as workforce shortages directly affected service delivery, outreach activities, information systems, and program monitoring.

#### 4.2.3 Health Information Systems

The study demonstrated that weaknesses in routine health information systems became more visible during the pandemic. Participants reported reduced availability of data capturers, delayed data entry, backlogs in reporting, and incomplete immunization records.

Importantly, respondents suggested that some apparent declines in immunization coverage reflected reporting and data‐quality challenges rather than actual reductions in service delivery. Similar concerns have been reported globally, where disruptions in routine health information systems complicated interpretation of immunization trends during COVID‐19 [20, 26].

The pandemic also highlighted the importance of robust routine monitoring and strengthened the recognition of strategic health information as a critical component of program management. Investment in electronic reporting systems and digital health infrastructure could strengthen preparedness for future emergencies [34].

#### 4.2.4 Essential Medicines, Vaccines and Technologies

Unlike reports from some countries where vaccine shortages disrupted routine immunization programs, vaccine availability was largely maintained throughout the study period [11, 15]. Managers attributed this resilience to effective provincial supply‐chain systems, including the Central Distribution Unit, stock redistribution mechanisms, and routine stock monitoring.

The isolated stock‐outs that occurred were generally related to inventory management deficiencies rather than national supply shortages. Similar recommendations for strengthening facility‐level stock management and monitoring systems have been reported elsewhere [34, 35].

This finding indicates that while access to services was affected by movement restrictions and workforce shortages, vaccine availability itself was not a major barrier to immunization delivery in the Free State Province.

#### 4.2.5 Health Systems Financing

Although financing was not identified as a major challenge by participants, several findings suggest underlying resource constraints. Respondents highlighted shortages of information technology infrastructure, connectivity problems, and inadequate digital tools for data management.

These findings illustrate that health‐system financing extends beyond the procurement of vaccines and includes ensuring adequate resources for digital infrastructure, monitoring systems, and emergency preparedness [18, 36]. The pandemic exposed existing infrastructure limitations and emphasized the need for strategic investments that enhance system resilience.

#### 4.2.6 Leadership and Governance

Leadership and governance emerged as both facilitators and barriers during the pandemic response. Participants acknowledged the value of partnerships with private healthcare providers, development partners, and district health teams. However, they also identified delayed communication, uncertainty regarding operational guidance, and inadequate emergency preparedness as major weaknesses.

These findings are consistent with previous studies examining South Africa's COVID‐19 response, which highlighted the importance of strong governance, effective communication, and preparedness planning [22, 37]. Managers described the response as largely reactive rather than guided by established emergency‐preparedness frameworks.

The findings suggest a need for stronger pandemic preparedness planning, including contingency staffing arrangements, continuity‐of‐service plans, and improved communication systems across all levels of the health sector.

#### 4.2.7 Risk Communication and Community Engagement

Risk Communication and Community Engagement (RCCE) emerged as an important cross‐cutting theme. Participants described how fear, misinformation, and inconsistent messaging negatively influenced healthcare‐seeking behavior. Several respondents reported that caregivers confused routine childhood immunization with COVID‐19 vaccination, leading to reluctance to attend healthcare facilities.

These findings are consistent with evidence demonstrating the critical role of communication and social mobilization in sustaining immunization demand [19, 38, 39, 40]. Poor communication and misinformation have previously been identified as barriers to vaccine uptake and contributors to vaccine hesitancy [39].

Participants highlighted community education, virtual messaging platforms, targeted outreach campaigns, and harmonized communication strategies as valuable approaches for maintaining demand during public health emergencies. Strengthening RCCE should therefore be considered an essential pillar of immunization programs and emergency preparedness planning.

#### 4.2.8 Implications for Future Pandemic Preparedness

The findings demonstrate that maintaining routine immunization services during public health emergencies requires health‐system resilience across multiple domains. The pandemic exposed weaknesses in workforce capacity, health information systems, emergency preparedness, and communication structures, while simultaneously revealing strengths in vaccine supply management and program adaptability.

Future preparedness efforts should focus on strengthening workforce capacity, investing in digital health infrastructure, institutionalizing catch‐up vaccination strategies, improving emergency communication systems, and integrating RCCE into routine program planning [26, 36, 38, 40]. Such investments are likely to improve the resilience of immunization programs while protecting essential health services during future emergencies.

#### 4.2.9 Study Limitations

This study has some limitations. First, the quantitative analysis relied on routinely collected DHIS data, which may have been affected by incomplete reporting and data quality issues. Qualitative findings suggested that COVID‐19‐related disruptions, including staff shortages and increased workloads, may have affected reporting completeness during the pandemic, potentially influencing the observed non‐significant decline in immunization uptake. Second, the study was conducted in the Free State Province, which may limit the generalizability of the findings. Third, the qualitative findings were based on healthcare workers' and managers' perspectives and may be subject to recall or social desirability bias. Finally, caregiver perspectives were not included, limiting insights into demand‐side barriers to childhood immunization. Despite these limitations, the mixed‐methods approach provided valuable insights into the impact of COVID‐19 on childhood immunization services.

## 5 Conclusion

The COVID‐19 pandemic disrupted access to and uptake of early childhood immunization services in the Free State Province, South Africa. Quantitative findings showed declines in immunization service utilization, while qualitative findings identified barriers related to movement restrictions, fear of infection, staff shortages, and service disruptions. Despite these challenges, healthcare facilities implemented adaptive strategies to maintain service delivery. Strengthening health system resilience, emergency preparedness, and continuity of routine immunization services is essential to protect child health during future public health emergencies.

## Author Contributions

**Abebe Sorsa Badacho:** writing – original draft, writing – review and editing, supervision, methodology, conceptualization, visualization, formal analysis, software, data curation. **Nomvuyiseko Maduna:** conceptualization, investigation, funding acquisition, methodology, validation, visualization, software, formal analysis, project administration, data curation, resources, writing – original draft, writing – review and editing. **Juliet Nyasulu:** conceptualization, investigation, funding acquisition, writing – original draft, writing – review and editing, methodology, visualization, validation, software, formal analysis, supervision.

## Funding

The authors have nothing to report.

## Ethics Statement

The study was part of a broader research project on maintaining essential services during the COVID‐19 pandemic, approved by the University of the Witwatersrand's HREC (Protocol #M200706). Ethical considerations included obtaining informed consent, maintaining confidentiality, and securing data. No incentives were offered, and participants were free to withdraw at any point. The researcher handled all processes from data collection to analysis to ensure data integrity.

## Conflicts of Interest

The authors declare no conflicts of interest.

## Transparency Statement

Corresponding author (A.S.B.), the manuscript guarantor, affirms that this manuscript is an honest, accurate, and transparent account of the study being reported; that no important aspects of the study have been omitted; and that any discrepancies from the study as planned (and, if relevant, registered) have been explained.

## Data Availability

The data that support the findings of this study are available from the corresponding author upon reasonable request.
